# Evaluating influence of the genotypes in the follicle-stimulating hormone receptor (FSHR) Ser680Asn (rs6166) polymorphism on poor and hyper-responders to ovarian stimulation: a meta-analysis

**DOI:** 10.1186/s13048-014-0122-2

**Published:** 2014-12-20

**Authors:** Noel Pabalan, Camila Martins Trevisan, Carla Peluso, Hamdi Jarjanazi, Denise Maria Christofolini, Caio Parente Barbosa, Bianca Bianco

**Affiliations:** Center for Research and Development, Angeles University Foundation, Angeles City, 2009 Philippines; Graduate School, Cebu Doctors’ University, Mandaue City, 6014 Philippines; Research and Extensions Office, Saint Louis University, BaguioCity, 2006 Philippines; Human Reproduction and Genetics Center, Department of Collective Health - Faculdade de Medicina do ABC, Av. Príncipe de Gales, 821, Santo André/SP, São Paulo Zip Code 09060-650 Brazil; Environmental Monitoring and Reporting Branch, Ontario Ministry of the Environment, 125 Resources Road, Etobicoke, ON M9P 3V6 Canada

**Keywords:** Follicle-stimulating hormone receptor, FSHR, Polymorphisms, Ovarian response, Meta-analysis

## Abstract

**Background/aims:**

Reported associations of controlled ovarian hyperstimulation response (COH) with genotypes of the Ser680Asn (N680S) polymorphism in the follicle stimulating hormone receptor (FSHR) gene have conflicting results.

**Methods:**

PubMed and Embase databases were searched for studies that investigated the N680S polymorphism in the FSHR gene in COH. Parameters used to examine ovarian response were poor and hyper-responses to COH. Using the meta-analytic approach, we estimated ovarian response risk (odds ratio [OR] with 95% confidence intervals) according to genotype.

**Results:**

Our findings showed that SS genotype carriers were most likely to be poor responders (OR 1.61, p = 0.08) compared to the NN and NS genotypes which showed no associations (OR 0.93-0.95, p = 0.75-0.78). Heterogeneity of these pooled ORs warranted examining its sources. We detected outlying studies in each of the three N680S genotypes. Omitting these outliers erased the heterogeneity of the recalculated pooled outcomes. It also materially altered the SS effects where carriers became slightly unlikely to be poor responders (OR 0.90, p = 0.52). The S allele carrier effect was modulated for poor responders (OR 1.24, p = 0.39) in the Non-Hispanic Caucasian (NHC) subgroup. The likelihood of the S allele carriers (OR 1.47, p = 0.02) and the unlikelihood of the N allele carriers (OR 0.64, p = 0.007) were significant in our hyper-response findings. Confined to NHC retained significance of the S allele effects (OR 1.57, p = 0.01) but not among the N allele carriers (OR 0.68, p = 0.18).

**Conclusions:**

In summary, this is a meta-analytical confirmation of the FSHR SS genotype role in COH response. Hyper-responder analysis strengths lie on the non-heterogeneity and robustness of its results. Non-robustness and heterogeneity of the poor-responder results compose its limitations. Thus, poor response findings probably require caution as to the interpretation as a susceptibility marker for ovarian response.

**Electronic supplementary material:**

The online version of this article (doi:10.1186/s13048-014-0122-2) contains supplementary material, which is available to authorized users.

## Background

*In vitro* fertilization (IVF) is a multi-step process involving collection of oocyte-containing follicles after controlled ovarian hyperstimulation (COH) with rFSH (recombinant Follicle-stimulation hormone). Further steps involve oocyte fertilization, embryo development, embryo transfer to the uterus, and implantation. All these steps are critical for successful IVF [1]. An initial critical step of this complex procedure is the COH, aiming to safely obtain an adequate number of high-quality oocytes, so as to allow selection of the most viable embryo for transfer [1,2]. However, women submitted to this procedure yield different numbers of oocytes. Poor responders provide no more than 4-5 oocytes. Those with 6-15 oocytes are normal responders and those with >15 oocytes are hyper-responders [3,4]. Poor responses result in retrieved oocytes reduced number and hyper-response may lead to ovarian hyperstimulation syndrome (OHSS). Poor response may warrant repeated stimulation cycles to obtain appropriate number of oocytes. In contrast, good response is modulated to avoid hyperstimulation [5]. Thus, the patients advanced identification will elicit these responses in order to standard treatment, which would be clinically beneficial. Several factors have been proposed to predict ovarian response. These are: age [6], hormonal status [7], cigarette smoking [8] and ovarian reserve [6,9]. Apart from these proposed predictors, polymorphisms in various genes, such as estrogen receptor alpha (ESR1), cytochrome P450 19A (CYP19A) and follicle-stimulating hormone (FSH) have been investigated as markers to predict ovarian response [1,10-13]. FSH has been implicated in follicular growth and maturation, granulosa cell proliferation and in estradiol/aromatase synthesis [14]. FSH effects are mediated by FSH receptor (FSHR), a G-protein-coupled receptor expressed in granulosa cells [15] which mediates FSH signal transduction through adenylate cyclase activation and elevation of intracellular cAMP [16]. The *FSHR* gene is located on chromosome 2p21 spanning a region of 54 kb [17] and contains one large exon, which encodes the transmembrane and intracellular domains; and nine smaller exons, which encode the extracellular domain [15]. Two non-synonymous SNPs have been identified in the coding region of exon 10 of the FSH receptor gene (http://www.ncbi.nlm.nih.gov/snp/?term=FSHR; GeneID: 2492; Locus tag: HGNC: 3969). The first (rs6165) is found within the extracellular domain (codon 307) in which A is substituted by G, changing codon 307 from threonine (ACT) to alanine (GCT). The second (rs6166) lies within the intracellular domain (codon 680), in which G is replaced by A. This leads to an amino acid change at position 680 from serine (AGT) to asparagine (AAT) [18]. These two SNPs are related to ovarian response and affect gene function by changing the e gene product properties and consequently modifying response to FSH [19]. These polymorphisms are in linkage disequilibrium (LD), resulting in the most frequent allelic combination of T307-N680 and A307-S680 [20,21]. Many previous studies including a recent meta-analysis [22] focused on the N680S polymorphism. Clinical studies have demonstrated that the N680S polymorphism determines ovarian response to FSH stimulation in patients undergoing IVF treatment [11,22,23].

Given the conflicting outcomes in the human reproduction investigations of the variants in the FSHR gene [17,24], we performed a meta-analysis to evaluate the role of the N680S *FSHR* polymorphism in ovarian response. Here, we compared the association of inactivating *FSHR* genotypes in (i) poor and (ii) hyper-responding women with normal ovarian response.

## Materials and methods

### Selection of studies

Using the terms, “FSHR”, “follicle stimulating hormone”, “*polymorphism*” and “*ovarian stimulation*”, we searched MEDLINE using PubMed and Embase for associated studies as of September 12, 2014. References cited in the retrieved articles were also screened manually to identify additional eligible studies. Inclusion criteria included: (1) case–control study design evaluating the association between FSHR polymorphisms and ovarian response, (2) sufficient genotype frequency data presented to calculate the odds ratios (ORs) and 95% confidence intervals (CIs). (3) Control frequencies (normal/good responders) must be in Hardy-Weinberg Equilibrium (HWE).

### Data extraction

Two investigators, independently, extracted data and reached consensus on all the items. The following information was obtained from each publication: first author’s name, published year, country of origin, dominant ancestry of the study populations, study design, context of the study, type of ovarian response, use of the HWE, addressed LD, FSHR polymorphism studied, sample source, genotyping approach, matching information and genotype data. We extracted data that belonged to our investigation of ovarian response using these parameters: (i) number of poor and (ii) hyper-responders as well as (iii) normal (good/control) responders. We also calculated frequencies of the variant allele, deviations of the normal responders from the HWE. (iv) Rates of pregnancy.

### Quality assessment of the studies

We used the Clark-Baudouin Score (CBS) to evaluate the methodological quality of the included studies [25]. This scale emphasizes statistics (i.e. p values, power and corrections for multiplicity) and includes genotyping methods as well as HWE. These, among others, were addressed in several of the included papers. Thus, we felt this to be the most appropriate in assessing the methodological included studies quality. The CBS scores reach from 0 (worst) to 9 (best) for cohort studies and 0 (worst) to 10 (best) for case-control studies. The score follows as: case-control studies: (quality is low for <5 and high for ≥5); cohort studies: (quality is low for <4 and high for ≥4).

### Meta-analysis

Due to the FSHR polymorphisms (T307A and N680S) were found to be in complete LD [20,21], we only investigated the N680S polymorphism. Influence of FSHR N680S polymorphism was estimated using the following parameters: we examined (i) poor ovarian response, (ii) hyper-response compared to the (iii) normal or good responders using the homozygote variant and wild-type alleles as well as the heterozygote genotype. We also examined (iv) pregnancy rates. These associations were expressed as OR, 95% CI.

Raw data for genotype frequencies, without adjustment, were used for calculating OR study-specific estimates. The pooled estimates significance was determined by the Z-test. Pooled estimates were obtained using either the fixed [26] (in the absence of heterogeneity) or random [27] (in its presence) effects models. Heterogeneity between studies was estimated using the χ^2^-based Q test [28]. Recognizing the low power of this test [29], significance threshold was set at p = 0.10. We also quantified heterogeneity with the I^2^ statistic which measures the degree of inconsistency among studies [30]. Significance was set at a p-value of ≤0.05 throughout, except in heterogeneity estimation. Pooled estimates were submitted to sensitivity analysis which involved omitting one study at a time and recalculating the pooled estimates, to test the summary effect robustness. Subgroup analysis based on ethnicity was performed on those comprising of three or more subgroups. Data were analyzed using Review Manager 5.3 (Copenhagen: Nordic Cochrane Centre, Cochrane Collaboration, 2014) and SigmaStat 2.3 (Systat Software, San Jose, CA). Most comparisons in our meta-analysis had less than 10 studies, and because of the low sensitivity of the qualitative and quantitative tests [31], we did not investigate publication bias. However, the overall analysis of poor responders had 11 studies; for those, we used Begg’s test to evaluate whether the magnitude of the observed association was related to the variance of each study [32].

## Results

### Included studies

**Figure 1:**
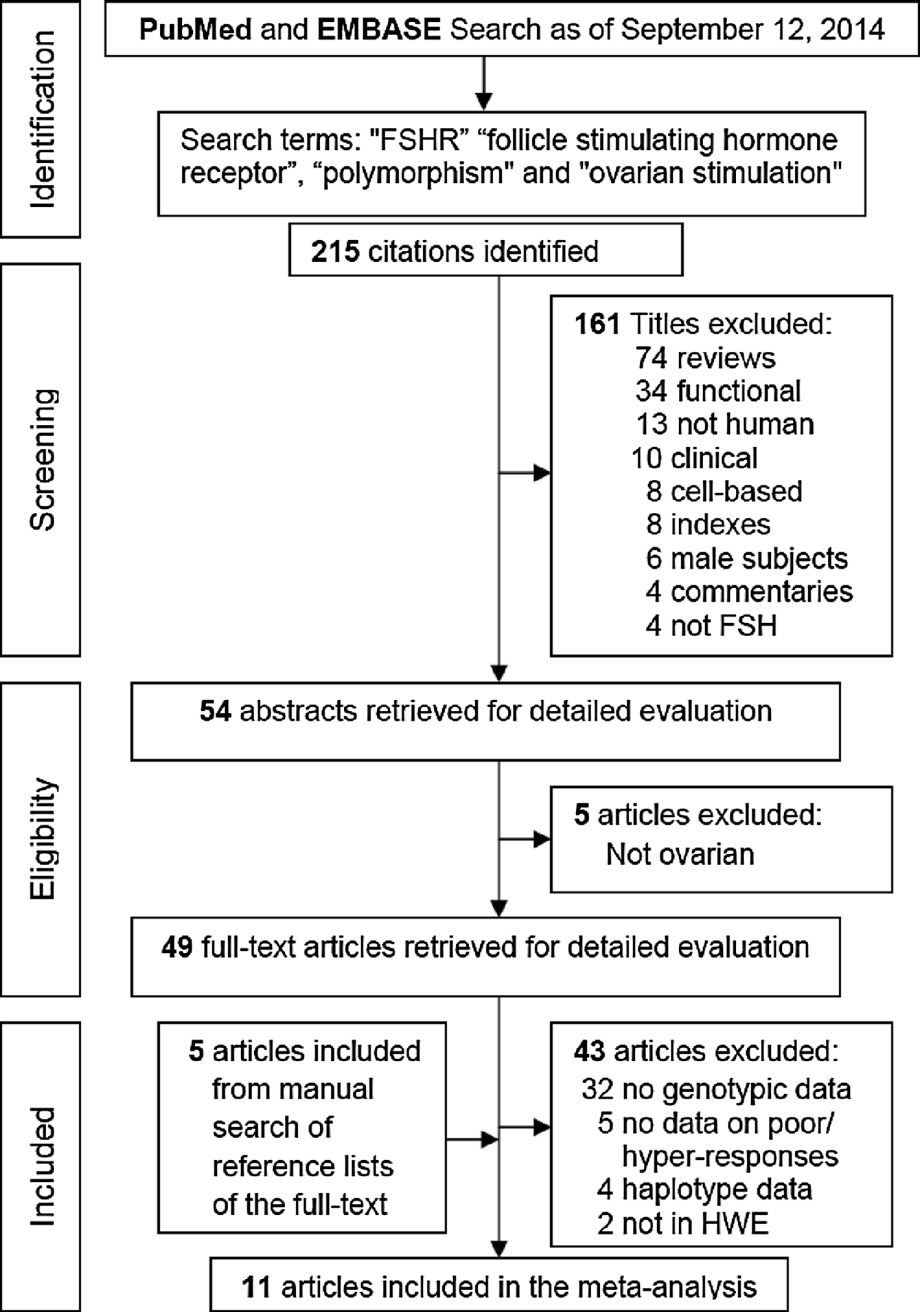
**Flowchart of literature search and study selection.**

Figure 1 outlines our study selection process in a flowchart following PRISMA (Preferred Reporting Items for Systematic Reviews and Meta-Analyses) guidelines [33]. We identified a total of 215 citations during the initial search, from which 161 were omitted because they were not conforming our inclusion criteria. We retrieved the remaining 54 abstracts which after review, five articles were excluded. Full-texts of the remaining 49 were obtained and read including the reference lists. From these lists, we found five articles with potential inclusion in the meta-analysis. From the 54 full-text articles, 43 were removed, including two studies [3,34] which control frequencies deviated from the HWE. Under these circumstances, the total number of articles included in the meta-analysis was 11 [1,4,13,35-42]. This number was increased to 13 studies, due to the provision of the independent data added from Boudjenah et al [1] and Livshyts et al [39].

The epidemiological features and clinical characteristics of these studies are outlined in Table 1. The publication years of the included studies ranged from 2003 to 2014. Six studies [1,36,38-40,42] had subjects that were of non-Hispanic Caucasian (NHC) descendant, two [13,37] were Hispanic Caucasian (HC) and three were Asian [4,35,41]. Eight of each 11 articles (72.7%) addressed HWE and LD, respectively. Based on the CBS scores, methodological quality of the included papers was high (mean and standard deviation of 5.82 ± 1.3).

**Table 1:** **Included articles characteristics that examined the FSHR N680S polymorphism association with ovarian response**

**First author**	**PY**	**[R]**	**Country**	**Ethnic group**	**Sample size**	**Type of ovarian response**	**Used HWE**	**Addressed LD**	**Tissue source**	**Genotyping**	**CBS**
Daelemans	2004	[36]	Belgium	NHC	229	Hyper/controls	No	Yes	blood	DNA sequencing	4
Klinkert	2006	[38]	Nether-lands	NHC	105	Poor, not poor	No	Yes	blood	Taqman	5
Livshyts	2009	[39]	Ukraine	NHC	374	poor, good/controls	Yes	Yes	blood	PCR-RFLP	5
Boudjenah	2012	[1]	France	NHC	427	poor, hyper/controls	Yes	Yes	blood	Multiplex PCR	7
Binder	2012	[42]	Germany	NHC	259	low, control	No	No	blood	RT-QPCR	6
Mohiyiddeen	2013	[40]	United Kingdom	NHC	504	poor, normal, hyper	Yes	Yes	blood	Taqman	5
de Castro	2003	[37]	Spain	HC	102	poor, good	Yes	Yes	blood	RT-QPCR	5
de Castro	2004	[13]	Spain	HC	170	poor, rest of patients, high	Yes	No	blood	RT-QPCR	8
Huang	2014	[4]	China	Asian	1,250	Poor, good responders	Yes	Yes	blood	MALDI-TOF	8
Achrekar	2009	[35]	India	Asian	150	Hyper, not hyper	Yes	No	blood	RT-QPCR	5
Yan	2013	[41]	China	Asian	450	Poor, not poor, hyper, not hyper	Yes	Yes	blood	RT-QPCR	6

PY: Publication Year; [R]: Reference; NHC: Non-Hispanic Caucasian; HC: Hispanic Caucasian; HWE: Hardy-Weinberg Equilibrium; LD: Linkage Disequilibrium PCR: Polymerase Chain Reaction; RFLP: Restriction Fragment Length Polymorphism; RT-QPCR: Real-Time Quality Polymerase Chain Reaction; MALDI-TOF: Matrix Assisted Laser Desorption Ionization-Time-of-Flight; CBS: Clark-Baudouin Score: assessment of methodological quality of the included studies.

Additional file 1: Table S1 shows the 11 studies from nine articles [1,4,13,35,37-42] summarizing the genotype frequencies of the poor responders compared to normal/good responders. Additional file 2: Table S2 displays the six studies from five articles [1,13,35,36,40] summarizing genotype frequencies of the hyper-responders compared to normal responders.

### Overall and subgroup analysis

**Figure 2:**
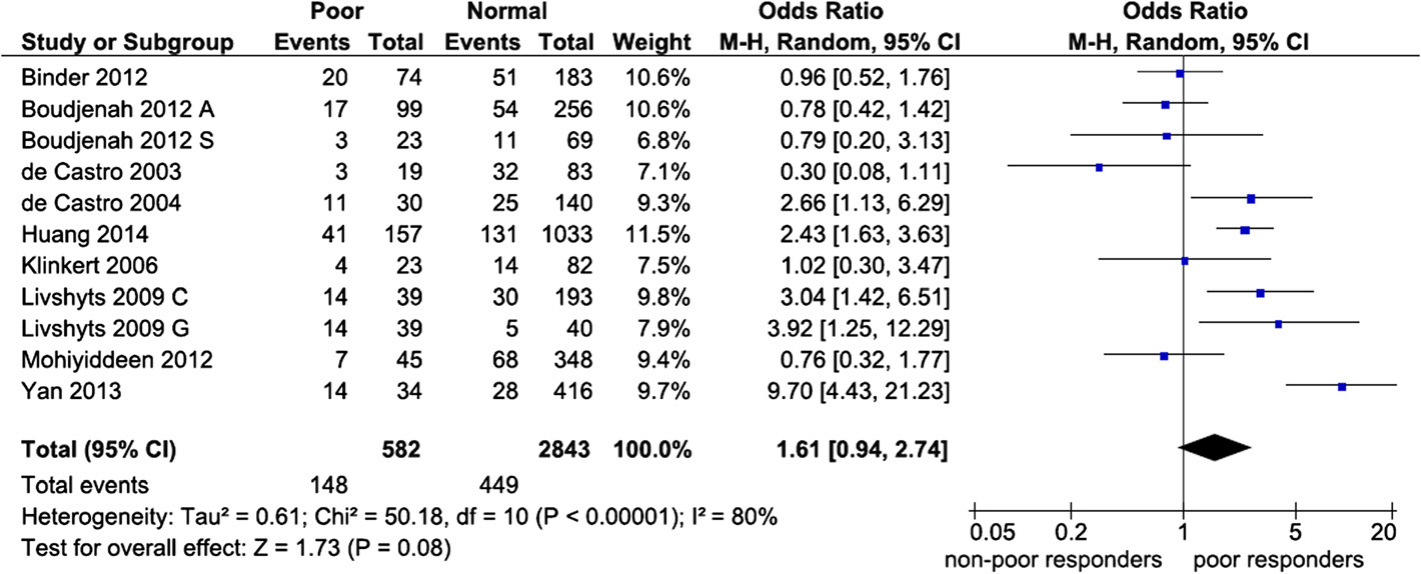
**Forest plot of the S680 influence variant on poor ovarian response.** Black diamond denotes the pooled OR. Blue squares indicate the OR in each study, with square sizes directly proportional to the weight contribution (%) of the study. Horizontal lines represent 95% confidence intervals. Note: In Boudjenah et al. [1], the suffixes A and S indicate overall population and homogeneous subgroup, respectively; In Livshyts 2009, the suffixes C and G indicate control women under 35 years old and over 35 years old, respectively. M-H: Mantel-Haenszel; CI: confidence interval; df: degree of freedom.

**Figure 3:**
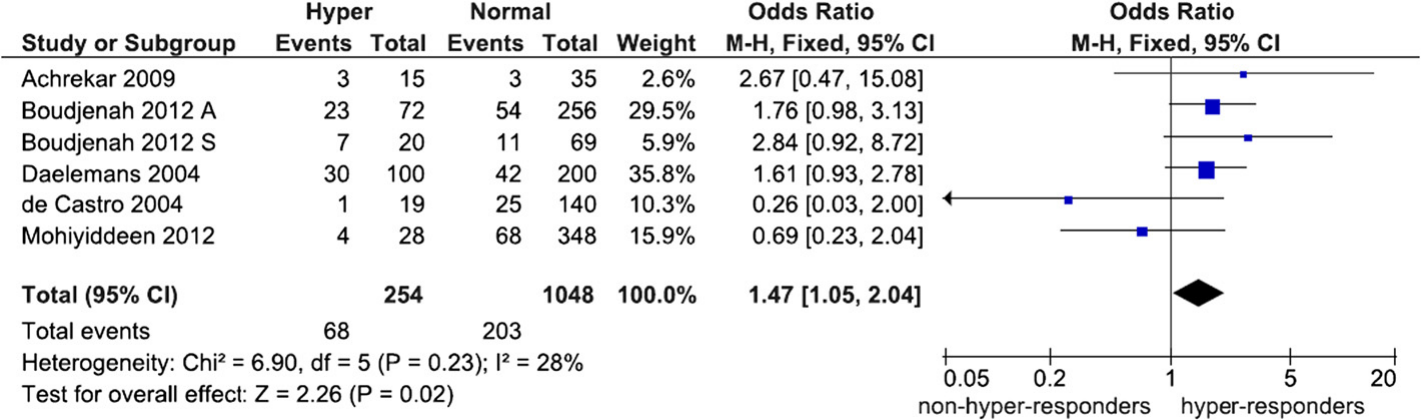
**Forest plot of the S680 influence variant on hyper ovarian response.** Black diamond denotes the pooled OR. Blue squares indicate the OR in each study, with square sizes directly proportional to the weight contribution (%) of the study. Horizontal lines represent 95% confidence intervals. Note: In Boudjenah et al. [1], the suffixes A and S indicate overall population and homogeneous subgroup, respectively; . M-H: Mantel-Haenszel; CI: confidence interval; df: degree of freedom.

We found no evidence of publication bias in the overall analysis of poor responders (p = 0.39-0.94). Table 2 and Figures 2 and 3 show an overall favoring of the SS genotype among the ovarian response groups, significant for hyper-responders (OR 1.47, p = 0.02) but not for poor responders (OR 1.61, p = 0.08). Confined to NHC modulated the poor responder effects (OR 1.24, p = 0.39) but not the hyper-responder effects (OR 1.57, p = 0.01). The N allele carriers unlikelihood of being ovarian responders was exemplified among hyper-responders with an overall significance (OR 0.64, p = 0.007). Of the studies listed in Table 1, six reported pregnancy rates [3,34,36,38,40,43], half of these had controls that deviated from the HWE [3,34,36]. All pregnancy rates pooled effects indicated absence of associations (data not shown).

**Table 2:** **Summary of the FSHR N680S polymorphism with poor and hyper-responders**

		**S allele**						**N allele**						**NS genotype**					
		**Test of association**			**Test of heterogeneity**			**Test of association**			**Test of heterogeneity**			**Test of association**			**Test of heterogeneity**		
	**N**	**OR**	**95% CI**	**pA**	**pB**	**I** ^**2**^	**AM**	**OR**	**95% CI**	**pA**	**pB**	**I** ^**2**^	**AM**	**OR**	**95% CI**	**pA**	**pB**	**I** ^**2**^	**AM**
Poor responders																			
All	11	1.61	0.94-2.74	0.08	<0.0001	80	R	0.95	0.66-1.37	0.78	0.002	65	R	0.93	0.60-1.46	0.75	<0.0001	80	R
Outliers omitted	--	0.90	0.66-1.23	0.52*	0.16	36	F	1.02	0.82-1.26	.88**	0.10	42	F	0.82	0.66-1.02	.08***	0.78	0	F
NHC	7	1.24	0.76-2.05	0.39	0.03	57	R	0.99	0.60-1.62	0.97	0.009	65	R	0.88	0.69-1.13	0.32	0.79	0	F
Hyper-responders																			
All	6	**1.47**	**1.05-2.04**	**0.02**	0.23	28	F	**0.64**	**0.46-0.88**	**0.007**	0.19	32	F	1.10	0.83-1.46	0.50	0.37	7	F
NHC	4	**1.57**	**1.11-2.23**	**0.01**	0.33	13	F	0.68	0.38-1.20	0.18	0.07	57	R	1.03	0.76-1.40	0.83	0.69	0	F

NHC: Non-Hispanic Caucasian; N: number of studies; OR: odds ratio; CI: confidence interval; pA: p value for association with significance set at <0.05; pB: p value for heterogeneity with significance set at <0.10; I^2^ values as measure of heterogeneity are considered low (<44%), moderate (45-74%) or high (>75%); values in **bold** indicate significant associations; *N = 7; **N = 8; ***N = 10; AM: Analysis Model; R: Random-effects; F: Fixed-effects.

### Outlier analysis

**Figure 4:**
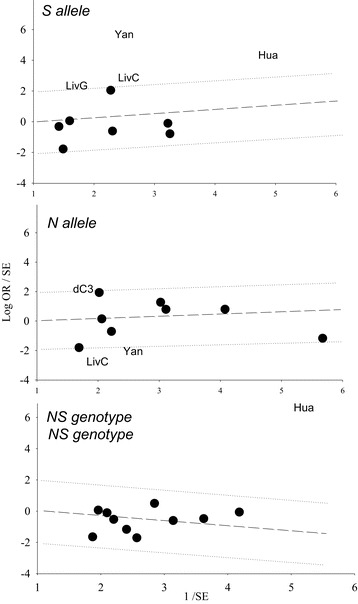
**Galbraith plot analysis to detect sources of heterogeneity.** LivC: Livshyts C; LivG: Livshyts G; dC3: de Castro et al. [37]; Hua: Huang; Note: In Livshyts, the suffixes C and G indicate control women less than 35 years old and over 35 years old, respectively.

We sought outlier studies as sources of the heterogeneity observed in the overall poor responder pooled effects (Figure 2). The Galbraith plots in Figure 4 show the outliers, four studies from three articles [4,39,41] in the S allele, three studies [37,39,41] in the N allele and one study [4] in the NS genotype. Table 2 shows the results of removing these outliers followed by re-analysis which erased heterogeneity (p values for heterogeneity from < 0.0001 to = 0.78) in all three genotypes and altered the SS genotype effect (OR 0.90, p = 0.52).

### Sensitivity analysis

Table 3 summarizes the changes in association resulting from sensitivity treatment. These changes have affected the poor responder group more than the hyper-responders. Generally, finding of no associations have been altered to the likelihood of N allele carriers to be poor responders with omission of three studies from two articles [39,41]. This pattern was also observed in hyper-responders where no associations were changed to likelihood of NS carriers to be non-hyper-responders with omission of Daelemans et al [36].

**Table 3:** **Summary of sensitivity analysis findings**

**Original summary effects**				**Resulting pooled OR**		
**OR**	**95% CI**	**Nature of association**	**Omitted study [ref]**	**OR**	**95% CI**	**Effect of study omission**
*NN genotype poor response overall*						
0.95	0.66-1.37	no associations	Livshyts C [39]	1.05	0.75-1.48	slight likelihood of N carriers being poor responders
0.95	0.66-1.37	no associations	Yan [41]	1.03	0.70-1.50	slight likelihood of N carriers being poor responders
*SS genotype poor response NHC*						
1.24	0.76-2.05	S carriers likely to be poor responders	Livshyts C [39]	0.98	0.71-1.36	no associations
*NN genotype poor response NHC*						
0.99	0.60-1.62	no associations	Livshyts C [39]	1.23	0.93-1.64	likelihood of N carriers being poor responders
0.99	0.60-1.62	no associations	Livshyts G [39]	1.12	0.69-1.82	likelihood of N carriers being poor responders
*NS genotype hyper-response NHC*						
1.03	0.76-1.40	no associations	Daelemans [36]	0.90	0.60-1.34	slight likelihood of NS carriers being non-hyper-responders

OR: Odds ratio; CI: Confidence Interval; Note: In Livshyts 2009, the suffixes C and G indicate control women less than 35 years old and over 35 years old, respectively.

## Discussion

With a sample size of 4,020 for the N680S FSHR polymorphism, our meta-analysis has shown that the FSHR gene genotype is important in determining ovarian response. The N680S polymorphism has been shown to be associated with poor and hyper ovarian responses to stimulation. From the three genotypes, the most and least likely to be poor and hyper-responders to ovarian stimulation are SS and NS carriers, respectively. SS genotype carriers favoring seem to merit higher confidence in the hyper-responder findings than those in poor responders for two reasons: (i) hyper-responder findings were generally not heterogeneous which the poor responder results were. (ii) Sensitivity treatment showed that poor responder effects were not as robust as those of the hyper-responders. To our knowledge, this is the first meta-analysis to address poor and hyper-responses as indicators of ovarian response. A recent meta-analysis [22] examined the number of retrieved oocytes and basal FSH levels based on eight studies, but not the parameters we used here, except pregnancy rates in which our findings did not materially differ.

The study-specific findings showed that women homozygous for the FSHR S680 variant were less likely to be low responders and more likely to be high responders [1]. In a Chinese study of infertile women, the polymorphisms T307A and N680S were associated with ovarian response to FSH, with the SS genotype having higher rates of poor response, but not with OHSS [41]. A retrospective study in IVF patients has shown an association between the presence of the S variant and poor responses to gonadotropin stimulation, suggesting that the S680 allele was associated with a diminished sensitivity to FSH [37]. The implication of S680 allele as a potential marker for predicting poor ovarian response has been countered with reports suggesting no N680S association with ovarian response [44]. It has been suggested that the subjects with the NS genotype are more associated with good response to FSH stimulation, whereas the subjects with SS and NN genotypes have a tendency to resist FSH stimulation and thus require more exogenous FSH for ovarian stimulation [45]. All these show a lack of consistency in these association studies outcomes. Here, then, enters the utility of our meta-analysis.

The observation that the allelic FSH receptor variants are FSH sensitivity determinants has lead to the hypothesis that certain genetic changes may fine tune the hormonal regulation of ovarian physiology, and these can be attractive markers for various clinical applications including optimization of the exogenous FSH dose in ART (Assisted Reproduction Technique) programs [20,46].

Approximately 20% of women undergoing ovarian stimulation in an IVF program respond poorly to gonadotropin treatment [47]. Such patients show low concentrations of serum estradiol, fewer mature oocytes and reduced pregnancy rates. The basis for low response to gonadotropin is not well understood. Diminished ovarian reserve and increased maternal age may be associated with poor ovarian response [6]. Several parameters such as poor perifollicular flow [48], presence of ovarian autoantibodies [49] and day 3 serum FSH concentrations [50,51] have been proposed as ovarian response predictors, but have not been proven. In some poor responders, increasing the FSH dose may not help in achieving an increase in serum estradiol concentrations [52].

Level of FSHR expression also impacts greatly on the extent of FSH action. FSHR plays a fundamental role in determining the physiologic FSH responsiveness in the ovary. Studies suggest that reduced expression affects FSHR function thereby affecting folliculogenesis [53]. The FSHR reduced expression on granulosa cells has been shown to be associated with poor ovarian response [54]. The expression of FSH receptor and its ability to respond to the exogenous FSH seems to be a gonadotropin determinant treatment. Thus, altered FSH receptor expression and function seems to be a factor and may account for poor ovarian response. Recently it has been demonstrated that lower expression of the FSH receptor may account for poor ovarian response to gonadotropin stimulation, suggesting a critical role of FSH receptor in the ovarian response [54].

Interpreting these meta-analysis results would warrant awareness of its strengths and limitations. Strengths include: (i) all tissue sources were blood; (ii) most of the studies addressed the HWE and LD issues; (iii) consistency in identifying the SS genotype as most likely to be associated with ovarian response. (iv) Since we did not include studies which controls deviated from the HWE, we managed to minimize methodological weakness, such as biased selection of subjects, genotyping errors and population stratification [55].

On the other hand, there are a number of limitations in our study: (i) we did not address age as covariate of poor and hyper-response given the insufficiency of primary data from the included studies. (ii) genotyping approaches were not uniform, although half of the studies used real-time PCR (Polymerase Chain Reaction).

It is possible that the N680S polymorphism does not play any direct functional role in the development of OHSS, but it is in LD with other polymorphisms. It is also likely that variants in other than FSHR are relevant to ovarian response [56]. Variants in genes relevant to biochemical pathways involved in steroids production and folliculogenesis have been studied, but replication of these relationships has been lacking [13,57,58]. Genome wide association studies may be promising as did a recent one that found significant correlation with ovarian response [59]. It is conceivable that ovarian response related to any one locus will be small due to gene-gene as well as gene-environment interactions are likely to operate.

## Conclusion

Genotyping the FSHR N680S together with some additional markers may provide a means of identifying a group of poor responders before infertility treatment is initiated. Meanwhile, further studies regarding other SNPs (or haplotypes) in the FSHR gene may help better understand the role of FSHR gene and ovarian response. Since potential biases and confounders could not be ruled out completely in this meta-analysis, additional large case-control studies or later update meta-analysis may be warranted to validate or modify our findings. It would help that well-designed studies based on sample sizes commensurate with detection of small genotypic risks should allow more definitive conclusions about the association of FSHR polymorphisms and ovarian response.

## Additional files

Additional file 1: Table S1. Genotype frequencies of poor responders compared to normal/good responders. In Livshyts 2009, the suffixes C and G indicate control women under 35 years old and over 35 years old, respectively. In Boudjenah et al. [1], the suffixes A and S indicate overall population and homogeneous subgroup, respectively. NHC: Non-Hispanic Caucasian; HC: Hispanic Caucasian; maf: minor allele frequency; HWE: Hardy-Weinberg Equilibrium. Additional file 2: Table S2. Genotype frequencies of hyper-responders compared to normal responders. In Boudjenah et al. [1], the suffixes A and S indicate overall population and homogeneous subgroup, respectively; NHC: Non-Hispanic Caucasian; HC: Hispanic Caucasian; maf: minor allele frequency; HWE: Hardy-Weinberg Equilibrium.
