# Supplementary material for: Evaluating influence of the genotypes in the follicle-stimulating hormone receptor (FSHR) Ser680Asn (rs6166) polymorphism on poor and hyper-responders to ovarian stimulation: a meta-analysis
Source: J Ovarian Res. 2014 Dec 20;7:285. doi: 10.1186/s13048-014-0122-2 (PMC4279698; doi:10.1186/s13048-014-0122-2)
Supplement: Additional file 1: Table S1. — Genotype frequencies of poor responders compared to normal/good responders. In Livshyts 2009, the suffixes C and G indicate control women under 35 years old and over 35 years old, respectively. In Boudjenah et al. [1], the suffixes A and S indicate overall population and homogeneous subgroup, respectively. NHC: Non-Hispanic Caucasian; HC: Hispanic Caucasian; maf: minor allele frequency; HWE: Hardy-Weinberg Equilibrium. [file 13048_2014_122_MOESM1_ESM.doc]

**Supplementary Table S1** Genotype frequencies of poor responders compared to normal/good responders

|  |  |  | Poor response | | | | | Normal / Good | | | | |  |  |
| --- | --- | --- | --- | --- | --- | --- | --- | --- | --- | --- | --- | --- | --- | --- |
|  |  |  | Genotype | | | Allele | | Genotype | | | Allele | |  |  |
|  | First author |  | NN | NS | SS | N | S | NN | NS | SS | N | S | maf | HWE |
| 1 | Klinkert | NHC | 9 | 10 | 4 | 28 | 18 | 31 | 37 | 14 | 99 | 65 | 0.33 | 0.61 |
| 2 | Livshyts (G) | NHC | 5 | 20 | 14 | 30 | 48 | 72 | 91 | 30 | 235 | 151 | 0.39 | 0.88 |
| 3 | Livshyts (C) | NHC | 5 | 20 | 14 | 30 | 48 | 12 | 23 | 5 | 47 | 33 | 0.41 | 0.24 |
| 4 | Boudjenah (A) | NHC | 38 | 44 | 17 | 120 | 78 | 87 | 115 | 54 | 289 | 223 | 0.44 | 0.17 |
| 5 | Boudjenah (S) | NHC | 14 | 6 | 3 | 34 | 12 | 26 | 32 | 11 | 84 | 54 | 0.39 | 0.83 |
| 6 | Binder | NHC | 19 | 35 | 20 | 73 | 75 | 39 | 93 | 51 | 171 | 195 | 0.47 | 0.78 |
| 7 | Mohiyiddeen | NHC | 17 | 21 | 7 | 55 | 35 | 100 | 180 | 68 | 380 | 316 | 0.45 | 0.42 |
| 8 | de Castro 2003 | HC | 7 | 9 | 3 | 23 | 15 | 12 | 39 | 32 | 63 | 103 | 0.38 | 0.98 |
| 9 | de Castro 2004 | HC | 8 | 11 | 11 | 27 | 33 | 47 | 68 | 25 | 162 | 118 | 0.42 | 0.96 |
| 10 | Huang | Asian | 60 | 116 | 41 | 236 | 198 | 446 | 456 | 131 | 1348 | 718 | 0.35 | 0.39 |
| 11 | Yan | Asian | 10 | 10 | 14 | 30 | 38 | 201 | 187 | 28 | 589 | 243 | 0.29 | 0.08 |

In Livshyts 2009, the suffixes C and G indicate control women under 35 years old and over 35 years old, respectively.

In Boudjenah 2012, the suffixes A and S indicate overall population and homogeneous subgroup, respectively. NHC:

Non-Hispanic Caucasian; HC: Hispanic Caucasian; maf: minor allele frequency; HWE: Hardy-Weinberg Equilibrium
