# Supplementary material for: Evaluating influence of the genotypes in the follicle-stimulating hormone receptor (FSHR) Ser680Asn (rs6166) polymorphism on poor and hyper-responders to ovarian stimulation: a meta-analysis
Source: J Ovarian Res. 2014 Dec 20;7:285. doi: 10.1186/s13048-014-0122-2 (PMC4279698; doi:10.1186/s13048-014-0122-2)
Supplement: Additional file 2: Table S2. — Genotype frequencies of hyper-responders compared to normal responders. In Boudjenah et al. [1], the suffixes A and S indicate overall population and homogeneous subgroup, respectively; NHC: Non-Hispanic Caucasian; HC: Hispanic Caucasian; maf: minor allele frequency; HWE: Hardy-Weinberg Equilibrium. [file 13048_2014_122_MOESM2_ESM.doc]

**Supplementary Table S2** Genotype frequencies of hyper-responders compared to normal responders

|  |  |  | Hyper-response | | | | | Normal | | | | |  |  |
| --- | --- | --- | --- | --- | --- | --- | --- | --- | --- | --- | --- | --- | --- | --- |
|  |  | Sample size | Genotype | | | Allele | | Genotype | | | Allele | |  |  |
| First author |  |  | NN | NS | SS | N | S | NN | NS | SS | N | S | maf | HWE |
| Boudjenah (A) | NHC | 72 | 17 | 32 | 23 | 66 | 78 | 87 | 115 | 54 | 289 | 223 | 0.44 | 0.17 |
| Boudjenah (S) | NHC | 20 | 5 | 8 | 7 | 18 | 22 | 26 | 32 | 11 | 84 | 54 | 0.39 | 0.83 |
| Mohiyiddeen | NHC | 376 | 11 | 13 | 4 | 35 | 21 | 100 | 180 | 68 | 380 | 316 | 0.45 | 0.42 |
| Daelemans | NHC | 300 | 16 | 54 | 30 | 86 | 114 | 62 | 96 | 42 | 220 | 180 | 0.45 | 0.67 |
| de Castro 2004 | HC | 19 | 4 | 14 | 1 | 22 | 16 | 47 | 68 | 25 | 162 | 118 | 0.42 | 0.96 |
| Achrekar | Asian | 50 | 6 | 6 | 3 | 18 | 12 | 15 | 17 | 3 | 47 | 23 | 0.33 | 0.55 |

In Boudjenah 2012, the suffixes A and S indicate overall population and homogeneous subgroup, respectively; NHC: Non-Hispanic Caucasian; HC: Hispanic Caucasian; maf: minor allele frequency; HWE: Hardy-Weinberg Equilibrium
